# Female rats are more susceptible to central nervous system oxygen toxicity than male rats

**DOI:** 10.14814/phy2.282

**Published:** 2014-04-06

**Authors:** Heather E. Held, Raffaele Pilla, Geoffrey E. Ciarlone, Carol S. Landon, Jay B. Dean

**Affiliations:** 1 University of South Florida, 12901 Bruce B. Downs Blvd.MDC8, Tampa, 33612‐4799, Florida

**Keywords:** Central nervous system oxygen toxicity, decongestant, diving, hyperbaric oxygen, pseudoephedrine, sex differences

## Abstract

Tonic–clonic seizures typify central nervous system oxygen toxicity (CNS‐OT) in humans and animals exposed to high levels of oxygen, as are encountered during scuba diving. We previously demonstrated that high doses of pseudoephedrine (PSE) decrease the latency to seizure (LS) for CNS‐OT in young male rats. This study investigated whether female rats respond similarly to PSE and hyperbaric oxygen (HBO). We implanted 60 virgin stock (VS) and 54 former breeder (FB) female rats with radio‐telemetry devices that measured brain electrical activity. One week later, rats were gavaged with saline or PSE in saline (40, 80, 120, 160, or 320 mg/kg) before diving to five atmospheres absolute in 100% oxygen. The time between reaching maximum pressure and exhibiting seizure was LS. Vaginal smears identified estrus cycle phase. PSE did not decrease LS for VS or FB, primarily because they exhibited low LS for all conditions tested. VS had shorter LS than males at 0, 40, and 80 mg/kg (−42, −49, and −57%, respectively). FB also had shorter LS than males at 0, 40, and 80 mg/kg (−60, −86, and −73%, respectively). FB were older than VS (286 ± 10 days vs. 128 ± 5 days) and weighed more than VS (299 ± 2.7 g vs. 272 ± 2.1 g). Males tested were younger (88 ± 2 days), heavier (340 ± 4.5 g), and gained more weight postoperatively (7.2 ± 1.6 g) than either VS (−0.4 ± 1.5 g) or FB (−1.6 ± 1.5 g); however, LS correlated poorly with age, body mass, change in body mass, and estrus cycle phase. We hypothesize that differences in sex hormones underlie females' higher susceptibility to CNS‐OT than males.

## Abstract (precis)

We previously reported that high doses of pseudoephedrine (PSE), a decongestant commonly used by scuba divers, decreases latency to seizure in a male rat model of central nervous system oxygen toxicity (CNS‐OT). We have now found that PSE does not have the same effect in female rats (either virgin stock [VS] or former breeders [FB]), primarily because female rats exhibit an inherently low latency to seizure. This sex difference appears to be independent of age, body mass, and estrus cycle phase.

## Introduction

Pseudoephedrine (PSE) is an *α*‐ and *β*‐adrenergic agonist commonly used as a decongestant because it decreases nasal sinus inflammation and mucus production (Cohn 1965). In high doses, however, PSE can cause nonconvulsive seizures in humans (İsmailoğulları et al. 2011) and rats (Zhi and Levy 1990). PSE is commonly used by scuba divers (Taylor et al. 2002), which is significant because divers exposed to high partial pressures of oxygen may experience central nervous system oxygen toxicity (CNS‐OT), which manifests as grand mal seizures.

We previously demonstrated that high doses of PSE hydrochloride reduce the latency to seizure (LS) in a rat model of CNS‐OT (Pilla et al. 2013). That study was an important first step in understanding the degree of increased risk posed by high doses of PSE use in the context of diving; however, it included only young male animals, while the human recreational diving population is quite diverse. The most recently published edition of the Divers Alter Network's Annual Diving Report found that 40% of the dives reported were completed by women (Pollock et al. 2008). Additionally, both men and women participate in diving across a broad range of ages: from as young as 10 years to more than 70 years old (Pollock et al. 2008). Given that other seizure disorders show sex‐ and age‐related changes (Hauser 1992; Hauser et al. 1996), the lack of information regarding the effect of PSE on CNS‐OT in populations other than young males represents a significant knowledge gap and a potential barrier to safe human diving practices. The primary purpose of this study was to determine whether female rats exhibit a different response to PSE administration and HBO than male rats. We also sought to determine whether different phases of the estrus cycle altered susceptibility to CNS‐OT, as rats have shown greater long‐term potentiation during the proestrus cycle phase (Warren et al. 1995), which could predispose animals to seizure activity. Finally, while the body mass range used in our previous study limited our investigation of males to juveniles, the same body mass range encompassed the adult body weight for female Sprague Dawley rats, which allowed investigation into the effect of age on LS in females.

## Experimental Procedures

All experimental procedures related to this study were reviewed and approved by the University of South Florida's Institutional Animal Care and Use Committee (AAALAC Accredited #000434).This study was designed to mirror, as closely as possible, our previous work in male rats. Thus, with the exception of female‐specific procedures (i.e., estrus cycle determination), the methods and materials used here have been described before (Pilla et al. 2013). An overview is provided below.

### Study design

We initially intended to use a single cohort of female rats (250–350 g; Harlan Laboratories, Indianapolis, IN), which was split into six groups, each receiving either saline control or PSE dissolved in saline (40, 80, 120, 160, or 320 mg/kg body weight). These doses were the same as were used in our previous study of males (Pilla et al. 2013) and were initially chosen because 320 mg/kg body weight is half the dose that is lethal to 50% of animals (LD_50_), which would ensure that any effects of the drug would be obvious while still keeping the dose below a level that would be expected to induce seizures on its own. The lower doses were chosen as fractions of the highest dose in order to develop a dose–response curve.

During the course of the study, however, it became apparent that females identified as former breeders (FB) responded differently than those identified as virgin stock (VS). Thus, the study was expanded, and the rats were divided into two cohorts based on their reproductive history. Rats were divided into the planned six dosage groups with 9–10 animals per group. VS were assigned so that the study would contain an equal number of rats to be tested during each estrus cycle phase. While each group (each PSE dose) necessarily contained an unequal distribution of the four phases, there were no differences in the average dose of PSE received by rats in each cycle phase. Many of the FB rats did not exhibit estrus cycles, most likely due to their more advanced age, so no attempt was made to assess the effects of estrus cycles in FB.

Naïve animals were used to avoid the confounding, kindling effect that previous seizures may have on LS during subsequent exposures (Gaito 1976; McNamara et al. 1985; Pilla et al. 2012).

### Telemetry unit implantation and use

At the start of the study, under isoflurane anesthesia, sterile surgical techniques were used to implant rats with telemetry units (TA11CTA‐F40; Data Sciences International, St. Paul, MN) for the acquisition of electroencephalogram (EEG) recordings. The body of the module was placed intraperitoneally. The ends of the two leads were implanted through the skull on either side of midline, between lambda and bregma. Rats were provided subcutaneous carprofen analgesia at the time of surgical preparation and at 12 and 24 h postoperatively. Rats recovered for at least 7 days before exposure to HBO.

On the day of the experiment, rats were placed in a clear plexiglass housing, which was located on top of the telemetry receiver (DSI PhysioTel, model RPC‐1, St. Paul, MN) inside a hyperbaric chamber. The receiver was hard‐wired through the chamber wall to an acquisition interface unit outside the chamber. A video camera (AXIS 221 Network Camera, St. Paul, MN) was situated so that it recorded the rat's behavior through a viewing port in the chamber. The camera and the data acquisition interface unit were connected to a computer for real‐time data acquisition and storage.

### Estrus cycle phase determination

On the morning of the experiment, vaginal smears were made, which allowed for even distribution of cycle phases among groups. Early in the study, rats were randomly assigned among the PSE doses. As the study progressed, however, assignments were made based on which cycle phases were least represented in each dose group. In some instances, it was necessary to wait an additional day or more to allow rats to reach the required cycle phase.

Vaginal smears were prepared in the following manner: sterile cotton swabs were moistened with saline and introduced in to the vagina. Cells were gently removed from the vaginal walls by swabbing and were then transferred onto a clean glass microscope slide. Slides were examined at 4× and 40× magnification to identify cell morphology. Estrus cycle phase was determined using the criteria described by Marcondes et al. (2002). In summary, proestrus smears consisted primarily of round, nucleated epithelial cells; estrus smears consisted primarily of anucleate cornified cells; metestrus smears consisted of roughly equal proportions of epithelial cells, cornified cells, and leukocytes; and diestrus smears consisted primarily of leukocytes.

### Age determination

Most of the animals included in this study had their exact date of birth included in the information sent by the vendor. For these animals, the exact age in days was simply counted. Some animals, however, had only the month of birth listed. For these animals, the date of birth was assumed to be the 15th of that month, and the approximate age was calculated from that date. This was the case for the vast majority of FB (*n *= 49) but was true for only two VS. Four rats in the FB group did not have any record of the date of birth, and no attempt was made to estimate age for these animals.

### Pseudoephedrine preparation and administration

Each rat's dose of PSE was prepared on the morning of the experiment to minimize differences in hydration of the drug among animals. Powdered PSE (Sigma‐Aldrich, St. Louis, MO) was dissolved in 0.9% NaCl solution (Hospira, Lake Forest, IL). Each animal received 1 mL of either PSE dissolved in saline or saline only (control) via gavage.

### Diving profile and seizure determination

Following gavage, animals were placed, unrestrained, in the plexiglass housing in the hyperbaric chamber. The hyperbaric chamber was filled with air throughout the experiment to avoid an increased risk of fire. The plexiglass housing was initially flushed with air (10 min), then flushed with 100% O_2_ (15 min). The plexiglass housing and main chamber were then compressed in parallel at a rate of 0.7 atmospheres absolute (ATA)/min. Dives were timed so that the chambers reached 5 ATA 2 h after PSE was administered. This allowed sufficient time for blood levels of PSE to reach their peak (Kanfer et al. 1993).

**Figure 1.**
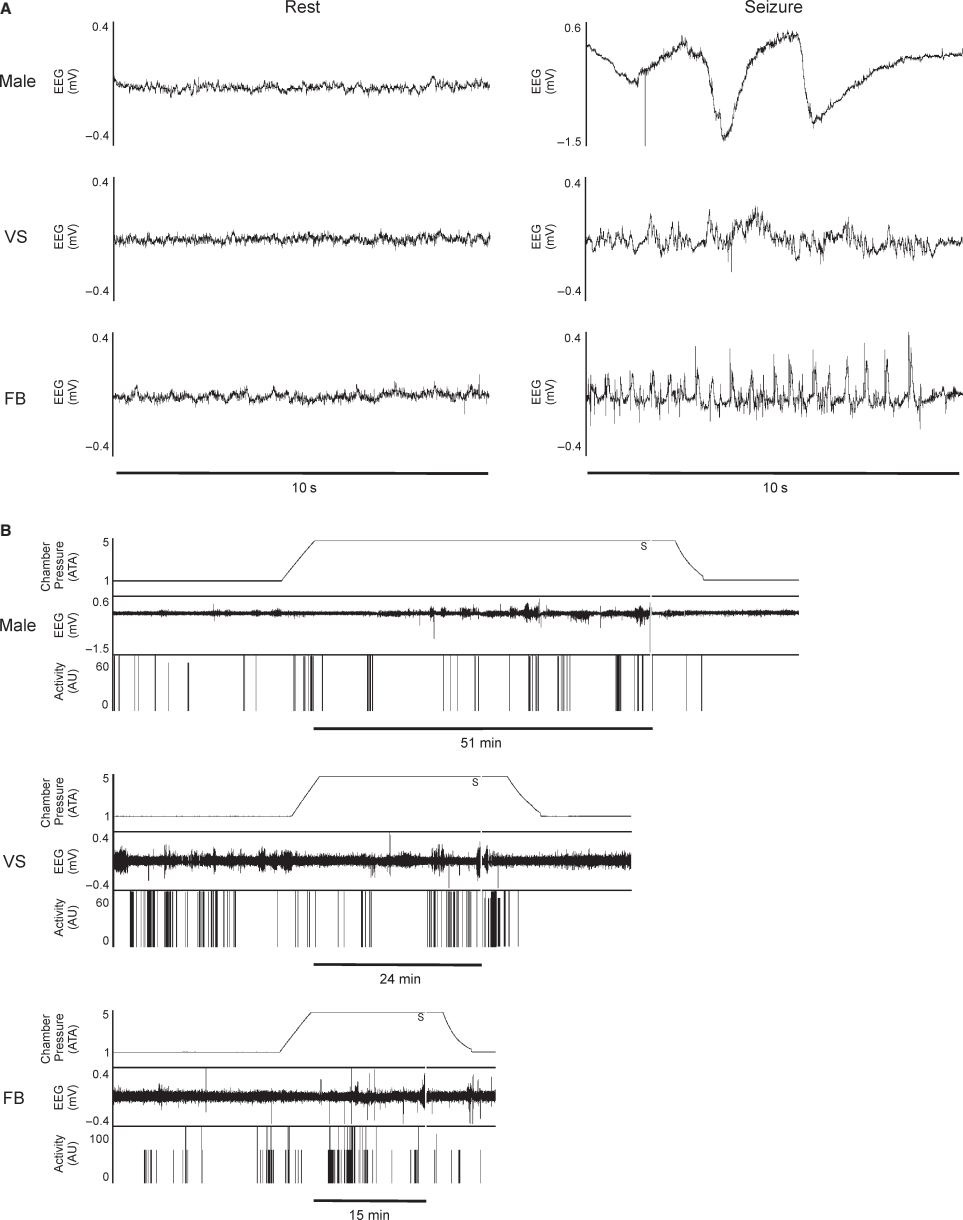
Representative data demonstrating changes in EEG with CNS‐OT onset from control (0 mg/kg PSE) animals. (A) Ten second examples of EEG recordings from normal (resting) state and during CNS‐OT seizure for Male, VS, and FB rats. EEG was always significantly elevated during tonic–clonic activity. (B) Whole experiment recordings showing chamber pressure, EEG activity, and animal activity (arbitrary units, AU). Onset of CNS‐OT seizure was defined as elevated EEG signal concurrent with tonic–clonic movements and is indicated by a vertical gray bar labeled with an S. LS was the time elapsed between reaching maximum pressure and onset of seizure. For each experiment, this is indicated with a horizontal black bar labeled in minutes.

Latency to seizure was the amount of time that elapsed between the chambers reaching 5 ATA oxygen and the rat exhibiting elevated EEG activity (Fig. 1A) concurrent with tonic–clonic movement of the head and forelimbs. Figure 1B shows representative data – including chamber pressure, EEG signal, and animal activity – for three animals in the control (0 mg/kg PSE) group. Immediately following seizure onset, the plexiglass chamber was flushed with air for approximately 3 min before both the plexiglass chamber and the outer chamber were decompressed to 1 ATA at a rate of 1 ATA/min. Rats were allowed to recover in air at 1 ATA for 15 min before being euthanized by CO_2_ overdose.

One VS rat in the 40 mg/kg dose group exhibited signs of pulmonary oxygen toxicity (gasping) prior to exhibiting seizure. This is rarely observed in either control or PSE animals, but has been observed in animals given ketone body precursors designed to extend LS (D'Agostino et al. 2013). For this rat, the dive was terminated at the time that gasping was observed, and decompression proceeded as described above. This animal was excluded from the dataset.

### Male results

**Figure 2.**
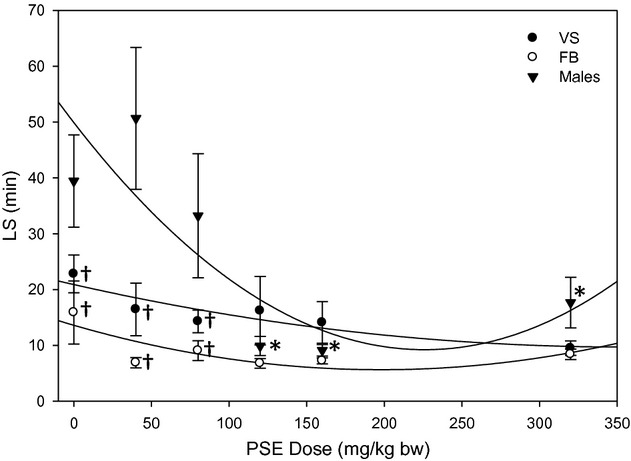
Latency to seizure as a function of PSE dose. LS correlated well with PSE dose for males (*n* = 54) and VS (*n* = 59). Sex differences were also apparent, as FB (*n* = 54) differed from males. ^*^indicates a significant reduction in LS compared to control (0 mg/kg),^ †^indicates a significantly lower LS than males at the same dose. Male data, included here for direct comparison with new data from females, are reproduced from Pilla R, Held HE, Landon CS, Dean JB (2013), “High doses of pseudoephedrine hydrochloride accelerate onset of CNS oxygen toxicity seizures in unanesthetized rats” *Neuroscience* 246:391–396, with permission from Elsevier.

Data for males in Fig. 2 (▾) are reprinted from Pilla et al. (2013) with permission from Elsevier.

### Data analysis

Data were analyzed using SigmaPlot 11.0. Effects of PSE dose and gender group (male, virgin stock female, or former breeder female) were assessed using a two‐way ANOVA. The Holm–Sidak method was used for pairwise multiple comparisons to determine specific differences among conditions. Similarly, effects of PSE dose and estrus cycle phase were evaluated using a two way ANOVA. LS was also plotted against age, body mass, and postoperative change in body mass in order to produce linear regressions to determine whether or not LS correlated with these other variables. Results were considered significant at *P* ≤ 0.05. All data are presented as mean ± standard error of the mean (SEM).

## Results

### Effect of PSE on LS

LS was plotted as a function of PSE dose (Fig. 2) for female rats (●, ○) and compared with data recently published (Pilla et al. 2013) for male rats (▾) under identical experimental conditions. As in male rats, LS in VS rats trended downwards as PSE dose increased, and a second‐order (nonlinear) curve fit the data better than a linear regression (*r*^2^ value for VS: 0.77 for a line, 0.83 for a curve; for FB 0.19 for a line, 0.64 for a curve). In females, the decrease in LS as a function of PSE was not significant; however, mean LS for both female groups appeared to be lower than for male rats, and the differences among groups were significant at the lower doses of PSE. VS exhibited lower LS than males at 0 mg/kg (*P* = 0.024), 40 mg/kg (*P* = 0.002) and 80 mg/kg (*P* = 0.019). FB exhibited lower LS than males at the control dose (*P* = 0.005), 40 mg/kg (*P* < 0.001), and 80 mg/kg (*P* = 0.004).VS and FB were not different from one another at any dose. Statistical power was high both for detecting differences based on PSE dose (0.977 for *α *= 0.05) and for detecting differences based on gender (1.000 for *α* = 0.05).

### Effect of age on LS

The effect of age on LS was investigated to determine whether these factors accounted for the differences between males and females. Males were the youngest cohort (88 ± 2 days), followed by VS (128 ± 5 days). FB were the oldest cohort (286 ± 10 days). Despite the age differences among groups, a linear regression revealed that LS did not correlate well with age per se (Table 1). This was true regardless of how the data were analyzed: with all rats combined, with males and females separated, with each of the cohorts (male, VS, FB) analyzed separately, by PSE dose, etc. Although females were categorized into VS and FB groups, female age was generally widely distributed, yet a linear regression still indicated that there was not a strong correlation between age and LS. The one trend that was present was a tendency for younger females to exhibit wider variability due to the presence of a relatively small number of animals with very long LS, while aged animals did not exhibit these outliers. LS for animals younger than 200 days (regardless of breeding status) was 15.6 ± 1.6 min, while those 200 days and older had an average LS of 8.33 ± 0.62 min (*P* < 0.001). The average PSE dose between these groups was not different. It is uncertain whether or not this trend exists in males, as all the males studied were under the age of 150 days.

**Table 1.** Effects of age, body mass, and body mass change on LS. Results of linear regressions are presented for male (*n* = 54), virgin stock female (*n* = 59), and former breeder female rats (*n* = 54), as well as regressions performed on the entire dataset (all three groups). Age, body mass, and body mass change did not correlate with LS for any of the groups individually, or when all data were combined.

	Males	VS	FB	All animals
Age	Slope: 0.30 min/day *r*^2^: 0.042	Slope: 0.05 min/day *r*^2^: 0.028	Slope: −0.03 min/day *r*^2^: 0.084	Slope: −0.05 min/day *r*^2^: 0.065
Body mass	Slope: 0.19 min/g *r*^2^: 0.069	Slope: 0.06 min/g *r*^2^: 7.87 × 10^−3^	Slope: −0.06 min/g *r*^2^: 0.026	Slope: 0.16 min/g *r*^2^: 0.091
Body mass change	Slope: −0.04 min/g *r*^2^: 4.27 × 10^−4^	Slope: −0.02 min/g *r*^2^: 6.93 × 10^−4^	Slope: 0.13 min/g *r*^2^: 0.038	Slope: 0.15 min/g *r*^2^: 8.57 × 10^−3^

### Effect of body mass on LS

Body mass was also measured and analyzed to determine whether it played a role in sex differences. Body mass differed among all of the groups (VS: 272 ± 2.1 g, FB: 299 ± 2.7 g, Males: 340 ± 4.5 g, *P* < 0.001 for all comparisons), which was somewhat surprising given that rats were purchased for the study by body mass. Despite these differences, body mass did not correlate with LS (Table 1).

### Effect of body mass change on LS

Finally, body mass change between surgery and the dive was investigated to see if it affected LS. Males tended to gain weight (7.2 ± 1.6 g), whereas VS and FB tended to remain at relatively stable body mass (−0.4 ± 1.5 g and −1.6 ± 1.5 g, respectively). There were significant differences between each of the female groups and the males (*P* < 0.001), but not between VS and FB. Despite the sex differences, change in body mass did not correlate with LS (Table 1).

### Effect of estrus cycle phase on LS

Estrus cycle phase was monitored in VS to determine if LS fluctuated by cycle phase. By design, there were no differences in mean PSE dose administered among the phases. Cycle phase did not significantly affect LS (Table 2), though the statistical power for this test was low (0.215 for *α* = 0.05).

**Table 2.** Effects of estrus cycle phase on LS. LS (mean ± SE) is presented for each estrus cycle phase of VS rats (*n* = 14 for diestrus, 15 for all other cycle phases). LS was not different among the estrus cycle phases.

Estrus cycle phase	Latency to seizure (min)
Proestrus	17 ± 3.2
Estrus	12 ± 2.6
Metestrus	13 ± 1.4
Diestrus	20 ± 4.8

## Discussion

### Study limitations

This study was designed primarily to test the effects of PSE administration in a female rat model of CNS‐OT. While estrus cycle phase served as a proxy for hormone levels, the lack of directly measured blood hormone levels precludes the possibility of directly correlating LS with hormones. In addition, it is possible that expanding the study to include two female cohorts (VS and FB) on the basis of early results could have biased the end results. This seems not to have been the case, though, given that ultimately no differences were found between VS and FB. Additionally, it should be noted that statistical power for identifying differences among estrus cycle phases was low. However, the standard deviation was quite high within each estrus cycle phase, so the lack of power is not surprising. Further study will likely be necessary to conclusively resolve this question.

### Effect of PSE on LS

Although the doses of PSE tested are substantially higher than those used by humans, we believe the doses are appropriate due to the effect of elevated rat metabolism on drug clearance. Rats' resting metabolic rate is approximately seven times greater than humans (Freireich et al. 1966) and results in drug metabolism that is roughly seven times that of humans. In addition, bioavailability of pseudoephedrine following oral administration in rats is roughly half that for humans (Palamanda et al. 2010). The highest dose tested was roughly 10 times the recommended human dose. As the highest dose represents half the LD_50_, we could be reasonably assured that any effects of PSE would be evident, but the dose was not high enough to cause seizure or death in the absence of HBO. It should be noted that high doses of PSE have been shown to cause cardiovascular complications (Akay and Ozdemir 2008; Fidan et al. 2013) and that these may affect LS by altering circulation or cerebral perfusion; however, we posit that this is simply an additional mechanism by which PSE may affect divers and does not confound interpretation of the results. Simply put, the purpose of this study was to determine if PSE alters susceptibility to CNS‐OT, regardless of the mechanism. The doses used in this study are further justified because the second major goal of the study was to compare female susceptibility to CNS‐OT with that of males – something that required utilizing the same doses as in our first study.

Previously, we reported that PSE in doses exceeding the recommended dose increased the risk for CNS‐OT in male rats (Pilla et al. 2013). This was not the case for female rats, most likely because females exhibited inherently shorter LS. It should be noted, however, that there was substantial variability among individuals, especially at the lower doses of PSE. These differences most likely reflect effects of the broad range of individual characteristics (e.g., age, body mass, etc.) included in the study and not any adverse effects of PSE. Given the lack of effect at low doses and the fact that PSE reduces the incidence of middle ear barotrauma (Brown et al. 1992), one of the most common diving‐related injuries (Pollock et al. 2008), there appears to be no reason at this time to limit normal use of the drug among divers.

### Effect of gender on LS

The fact that PSE did not reduce LS in females to the same extent as it did in males is likely due to the fact that females appear to exhibit lower innate LS for HBO toxicity than males. The difference between males and FB in the control group was particularly stark. This is a critical finding because it suggests that women, and in particular older women, may inherently be at higher risk for developing CNS‐OT. In addition to the implications for diving, our findings are also relevant to clinical practices where women may be exposed to high partial pressures of oxygen, such as during the course of hyperbaric oxygen therapy. This finding is even more important given that most diving tables and safe exposure limits were adapted from U.S. Navy diving practices, which were tested and developed around young men. It is difficult to assess how these findings translate to the real world, however, because CNS‐OT incidence is very low, with estimates of incidence around 2 per 100,000 dives in both clinical (Yildiz et al. 2004) and recreational settings (Pollock et al. 2008).

Although insufficient data exist to directly assess the role of gender in human CNS‐OT susceptibility, inferences can be made from comparisons with other seizure disorders. Studies in humans with epilepsy have elucidated potential roles for sex hormones in susceptibility to seizure. Men and women with epilepsy exhibit a higher incidence of endocrine disorders than the general population (Herzog et al. 1986a,b; Herzog 2002). Furthermore, recent work has indicated that endocrine disorders do not result directly from epilepsy or from antiepileptic drugs, as was previously thought (Bilo et al. 2001), which opens the possibility that endocrine dysfunction may be a contributing factor in epilepsy. In addition, it has been shown that epileptic women who enter menopause at a later age tend to have a lower frequency of seizures than women who are younger at the onset of menopause (Harden et al. 2003). Furthermore, it has been demonstrated that estrogen may act as a proconvulsant, while progesterone is anticonvulsant (Bäckström 1976; Frye and Rhodes 2005). In stark contrast, testosterone reduces seizure activity by altering neurosteroid levels (Reddy 2004). These studies demonstrate that sex hormones play an important role in normal brain function and offer insight into the mechanism by which different hormone levels affect susceptibility to seizure. We propose that the differences we observed among the groups in this study resulted primarily from the different levels of sex hormones present in young male, young female, and aged female rats. Further study will be required to confirm this hypothesis, however.

### Effect of age on LS

It should be noted that the differences in age among the groups tested are an artifact of the study design: as rats were selected for a specific body weight (based on the ideal range for implantation of the telemetry modules), males (which have a heavier adult body weight) were necessarily younger than females. Similarly, the vendor likely chose to ship the FB rats included in this study due to their decreased reproductive capacity, likely as a function of more advanced age. When comparing males with females, this is a difficult artifact to overcome; however, given that females were distributed across a broad range of ages and yet still showed no correlation between age and LS, it is reasonable to accept the null hypothesis (age alone does not directly affect LS).

Despite the age differences among groups, age correlated poorly with LS in our rats. These results are consistent with the findings from other seizure models, which show age‐dependent effects early in life, but little or no effect of age on LS in rats older than 60–80 days (Cavalheiro et al. 1987; Bough et al. 1999). We thus conclude that age alone does not explain the observed differences among groups and that some other underlying factor, such as hormone levels, must be responsible for the observed differences.

### Effect of body mass on LS

The differences in body mass among groups were surprising given that all rats were purchased using the same body weight criteria. The greater body mass for males is most likely due to the higher adult body mass for these animals and simply reflects their healthy growth at this age. Similarly, the VS females likely weighed a bit less than the FB rats because they were younger. Regardless, the lack of correlation between body mass and LS is not particularly surprising given that PSE doses were tailored to each rat's body mass. Thus, differences in body mass do not explain the observed sex differences in LS.

### Effect of body mass change on LS

Body mass change was studied for two reasons. First, it serves as a proxy for postoperative recovery. Those animals that recover well following surgery would be expected to maintain or increase their body weight, while excessive stress or poor postoperative recovery would be expected to decrease body weight. Second, recent work in our laboratory has indicated that elevating blood ketone levels delays the onset of CNS‐OT (D'Agostino et al. 2013). Animals that lose body mass likely metabolize some body fat, which could elevate blood ketones and increase LS.

### Effect of estrus cycle phase on LS

The fact that different estrus cycle phases did not appear to affect LS somewhat contradicts our supposition that sex hormones are responsible for differences among the groups. It is possible, however, that the difference in sex hormone levels among males, VS, and FB is much greater than the difference in levels among the cycle phases. The standard deviation for each group was fairly high, which diminished statistical power. At this time, it is difficult to determine whether the limited statistical power has resulted in a type 2 error, or if differences among estrus cycle phases are so small as to be unimportant when considering susceptibility to CNS‐OT. Future studies are needed to conclusively answer this question.

## Conclusions

Female rats, particularly FB, exhibited shorter LS than males when breathing 5 ATA oxygen. PSE did not reduce LS in females, even at doses that far exceeded recommendations. Age, body mass, and changes in body mass following surgery differed between males and females, but these differences did not correlate with alterations in LS and thus do not explain the sex differences identified in this study. The major finding from this study is that although females may not experience increased risk of CNS‐OT as a result of PSE use, they may have a higher innate risk for CNS‐OT than males. Given that this appears to be an effect of gender per se, women may want to take special care to remain within the established guidelines for safe diving when using gas mixtures and dive profiles that expose them to high doses of oxygen.

## Conflict of interest

None declared.
